# Impact of COVID-19 pandemic on the existence of social solidarity: evidence from rural-urban communities in Lombok Island, Indonesia

**DOI:** 10.3389/fsoc.2023.1164837

**Published:** 2023-05-05

**Authors:** Rosiady H. Sayuti, Moh Taqiuddin, Azhari Evendi, Siti Aisyah Hidayati, M. Zaenul Muttaqin

**Affiliations:** ^1^Sociology Study Program, University of Mataram, Mataram City, Indonesia; ^2^Faculty of Animal Husbandry, University of Mataram, Mataram City, Indonesia; ^3^Sociology Laboratory, University of Mataram, Mataram City, Indonesia; ^4^Faculty of Economics and Business, University of Mataram, Mataram City, Indonesia; ^5^Department of Public Administration, University of Cenderawasih, Jayapura City, Indonesia

**Keywords:** COVID-19 pandemic, Indonesia, rural, social solidarity, urban

## Abstract

The COVID-19 pandemic that has hit the entire world since the beginning of 2020 is an unimaginable phenomenon. The pandemic is disastrous because it has caused loss of life and livelihood for a large part of the population. People lose their jobs, spaces for social interaction are closed, and social relationships are disrupted. Several studies show that social solidarity should be a major concern for people to sustain the future quality of their lives. Social solidarity dimensions in this study include *gotong royong* (local culture of mutual help), marriage *banjars* (local association), cooperation, and sharing of information. This study aims to examine the existence of social solidarity during the pandemic in rural and urban areas and to know the level of community exposure to COVID-19 in Lombok Island, Indonesia. This research adopted a quantitative approach to identify and analyse the existence of social solidarity in rural and urban communities during the COVID-19 pandemic. A set of questionnaires was distributed and shared by enumerators with 1,100 targeted-respondents from Lombok Island. The survey was carried out from 14th October to 28th October 2021, that is, during a recovery period from the pandemic while restrictions implemented by the government were still in full force. Chi square statistical analysis was used to test whether there were differences in social solidarity between those who lived in rural areas and those who lived in urban areas. This research found the existence of social solidarity in both rural and urban communities during the pandemic. The level of social solidarity in rural areas is higher than in urban areas. While related to the number of those who were exposed and died, conditions were higher in rural areas than in urban areas. However, in terms of the death rate, the level of death rate in urban areas is higher than in rural areas. This condition indicates differences in the characteristics of rural and urban communities, which can be considered in implementing policies during a future pandemic. This research provides evidence for local governments in formulating policies with a social solidarity perspective by taking into account the different characteristics of rural and urban communities.

## 1. Introduction

The COVID-19 has turned out to be an unprecedented global health and socio-economic crisis since its emergence in early 2020 (Gostin and Hodge, 2020; Yu et al., 2021). However, it is more than a health crisis. It is also a humanitarian crises (Barneveld et al., 2020). People's everyday lives were and continue to be drastically changed by this pandemic due to restrictions imposed by physical distancing, working or learning from home, loss of work, as well as reduced socially contact with family and friends (Lupton and Willis, 2021). The UN (2020) has reported that over 2.2 billion people in the world unable to wash their hands regularly because of lack of access to safe water and 1.8 billion not able to keep physical distancing due to homelessness, low quality housing, and overcrowded housing. In Indonesia, the Social Monitoring and Early Response Unit (SMERU) Research Institute (SMERU, 2022) has traced the pandemic's socio-economic impacts on 12,216 nationally representative households across all 34 Provinces (Sarker et al., 2021).

The COVID-19 pandemic is disastrous because it has caused loss of life and livelihood for a large part of the population due to the social distancing policy. People lose their jobs, spaces for social interaction are closed, and social relationships are disrupted. This pandemic continues to cause problems in the economic, social, and even political fields in various parts of the world (Alam, 2021). In Durkheim's view, conditions of instability can force communities to agree with each other and share to lighten the burden so that they soon stabilize again. This mutual agreement and spirit of sharing are known as social solidarity (Alfirdaus et al., 2015). However, social solidarity cannot be activated automatically due to the complexity of the socio-cultural, economic, and political structures. Several preconditions are needed for solidarity to work, especially amid the pressure of an unstable situation due to a disaster. In some cases, disaster situations can strengthen community social solidarity even though, in a number of communities, the social solidarity actually weakens.

Socially, COVID-19 has altered relationship patterns between individuals due to physical or social distance implementation, isolated or suspended usual social activities (see Hosseinzadeh et al., 2022), and led many researchers to grapple with crucial issues about various aspects of social cohesion, especially social capital and social solidarity (Kittel et al., 2021; Negură et al., 2021). Social capital is considered one of the principal dimensions of social cohesion (Carter and Cordero, 2022). The current studies explored and examined the functioning of social capital in handling the negative consequences of the COVID-19 pandemic both at the micro and macro levels (Negură et al., 2021; Carter and Cordero, 2022; Tatarko et al., 2022). Social capital, which includes norms, social networks, trust, and mutual respect, has facilitated rural communities in Java areas-Indonesia in preventing and managing the impact of the COVID-19 pandemic (Rofieq et al., 2022; Primadata et al., 2023). In the public health context, social capital can be developed and maintained by postering and enhancing social solidarity or empathy between high-risk and low-risk groups (Wong and Kohler, 2020). To build community resilience during the Pandemic, Baraka (2021) found that social capital has played a significant role in forming social solidarity initiatives, as in Egypt cases. Referring to this series explanation, our article focuses on exploring social solidarity between urban and rural communities by enriching analysis using social capital's perspectives.

Stok et al. (2021) highlighted the relationship between disparities between regions and the severity of COVID-19 infection. In his studies in various countries, such as the United States, Sweden, and Brazil, it can be concluded that relatively poor areas have higher exposure and mortality rates than regions that are somewhat more developed or rich. Even in the United States, there are differences between racial groups of people, where the African-American group has a higher exposure level than European-Americans (Abedi et al., 2021; Chen and Krieger, 2021).

### 1.1. Social solidarity

In Durkheim's view, conditions of instability can force communities to agree with each other and share to lighten the burden so that they soon stabilize again. This mutual agreement and spirit of sharing are known as social solidarity (Alfirdaus et al., 2015). However, social solidarity cannot be activated automatically due to the complexity of the socio-cultural, economic, and political structures. Several preconditions are needed for solidarity to work, especially amid the pressure of an unstable situation due to a disaster. In some cases, disaster situations can strengthen community social solidarity even though, in a number of communities, the social solidarity actually weakens.

Social solidarity is believed to be synonymous with sharing, tolerance, mutual relief, and even a form of exchange in disaster situations (Alfirdaus et al., 2015). In line with this explanation, the practices of social solidarity in the COVID-19 pandemic situation are essential to be explained theoretically. Therefore, we analyse the quantitative data to demonstrate the applicability of the concept of social solidarity in the context of “vulnerability” or “disaster,” namely in the COVID-19 pandemic situation, by making comparisons between rural and urban communities. Previous studies related to social solidarity and the COVID-19 pandemic have not discussed much of the differences between villages and cities but have focused more on issues of gender inequality (Mishra and Rath, 2020; Prainsack, 2020), social disparities based on ethnicity, race, and socioeconomic position (Stok et al., 2021), as well as other economic and social impacts (Suryahadi et al., 2020; Mustafa et al., 2021). Socio-economic impacts can also be seen in research (Mustafa et al., 2021) in Malaysia, using an intergenerational perspective to review the collective memory of the second wave of the pandemic. In their research, Mustafa et al. (2021) explained that the younger generation, with an age range of 18–30 years, refers to a pandemic more than the older generation due to significant lifestyle changes. In contrast to the older generation, who tend to put lifestyle aside and prioritize the changes brought about by the pandemic. We argue that the social construction of social solidarity between the two types of people is still different in disaster situations.

The main question is how far has the COVID-19 Pandemic affected the differences in the construction of social solidarity between these two types of society? How do the two communities maintain their social solidarity existence in a situation of “pressure” due to the negative impact of the COVID-19 pandemic? How does social solidarity function in both societies in the context of the COVID-19 pandemic? In the sociocultural context of Lombok Island, our analysis also answers the question posed by Li (2012): does the context of social solidarity being discussed refer to the current situation or a better situation in the future? Then does this social movement occur in society universally? Or are there differences between rural and urban areas, particularly concerning policies set? On the other hand, the urgency of research using a social solidarity lens was triggered to complement other studies that reveal uncertainty of reference in the design of national policies (Ilham et al., 2021).

This research on social solidarity at the local or community-level is important because policies related to the pandemic, such as restricting people from leaving their places of residence, maintaining distance, and wearing masks, are factors that can reduce the sense of social solidarity among citizens (Taylor, 2019; Tiffany, 2020). In addition to the efforts made by the government to overcome the impact caused by the COVID-19 pandemic, various initiatives have also emerged from the community. Solidarity actions that have grown and taken root in the grassroots community are increasingly being tested in the midst of the COVID-19 pandemic. The existence of pandemic has strengthened the values of social solidarity inherent in the body of the Indonesian nation (Sayuti, 2020; Sayuti and Taqiuddin, 2020). In addition, various social movements, such as distributing free groceries to those affected and other philanthropic movement, have sprung up in society.

Durkheim developed the theory of social solidarity because he believes that the function of society works by itself in providing benefits to its members. This idea was developed as a response to the notion that social solidarity is not found in modern society when people tend to become more individualistic as stated by Spencer, Maine, and Tönnies (Durkheim, 1984). Tiryakian (1972) in Alexander and Smith (2008) defines social solidarity as a form of attachment between individuals in society, a source of consensual morality, and a way for society to create social order. Durkheim has provided a rationale for discussions about the workings of social solidarity in chaotic situations such as disaster events and extreme instability. A case of instability due to the outbreak of crime, violence, and disasters or crises in various forms will be able to encourage people one to another in order to normalize the situation. The extreme instability caused by the crisis triggered community members to jointly create a balanced situation again through social solidarity as an act of sharing responsibility. The pressure situation due to the COVID-19 Pandemic will encourage people to find glue for their social interactions to share and help each other (Durkheim, 1912).

An explanation of the roles between villages and cities in the context of this pandemic is also essential. According to Malatzky et al. (2020), cities must be seen as heterogeneous, multicultural places and sources of innovation. In contrast, the village is the opposite, as a location that is relatively homogeneous, simpler, and tends to be more resistant to various innovations. With the COVID-19 pandemic, the character of cities and villages is a factor that influences the speed of the spread of COVID-19, as well as efforts to prevent and cure it. The high level of population density and community activity in cities makes pandemics in cities spread faster. Theoretically, social distancing policies, for example, would be easier to implement in cities than in villages. As Larsen (2013) argument, a sense of togetherness to seek peace and kinship and a more comfortable natural atmosphere in the rural area. Included is a sense of solidarity among fellow citizens, which is very much needed in dealing with this pandemic.

Chan (2021) in Taliep et al. (2022) state that solidarity is marked by togetherness that occurs in thoughts, emotions, or actions and activities. It goes on to say that solidarity is at the core of collective action that transcends social and geographical boundaries. Social solidarity in this context is dynamic and arises when some members of the community face a difficulty; after that, a desire arises to help each other among members community (Douwes et al., 2018; Tomasini, 2020; Cho et al., 2021).

Taliep et al. (2022) explored social behavior and community solidarity in South Africa during the pandemic. The conclusion shows that, in general, it can be explained that the solidarity and social behavior of the community during the pandemic has materialized, regardless of their social and economic status. Other researchers (Tomasini, 2020; Taliep et al., 2021) they stated that throughout the world, there had been a sense of solidarity and prosocial behavior that had never happened before as a response to a pandemic with so many victims being exposed. In addition, many community members are taking part in efforts to help others during this pandemic, such as volunteering at existing health facilities or providing food assistance and supporting families exposed to COVID-19 (Sin et al., 2021; Taliep et al., 2021).

In their research on German society, Kaup et al. (2022) outlined the critical meaning of solidarity during a pandemic. He divides solidarity into three levels, namely institutional solidarity, group solidarity, and individual solidarity. They were first related to policies in dealing with a pandemic, such as the existence of a welfare or social security program. Both activities are associated with groups, such as using masks that impact other people. The third is someone's empathy for others who are exposed and who voluntarily help with their needs.

Brown (2020) explains that there is a relationship between solidarity between individuals and the level of public exposure to a pandemic. It is said that in societies where the level of solidarity is higher, the number of reported cases of exposure tends to be less. Furthermore, Kaup et al. (2022) further divided interpersonal solidarity, namely, solidarity received, and solidarity was shown. What is meant by solidarity received is how much or how often a person gets assistance during a pandemic. Meanwhile, what is meant by showing solidarity is how often someone assists those exposed during a pandemic. Finally, another researcher (Angaw, 2021) in research in Ethiopia concluded that institutional solidarity, in the form of social organizations that help people during a pandemic, has a massive role in reducing the impact caused by the large number of people exposed to the pandemic.

In his view, Stok et al. (2021) examine the challenges to solidarity that arise with this pandemic. This challenge then gave birth to three new types of solidarity: Intergenerational solidarity and cross-generational solidarity, where there must be a mutual understanding between the older and younger generations in dealing with a pandemic. Initially, the younger generation was asked to be more active in keeping their distance from the older generation. Later, the younger generation asked the older generation to reduce some of the policies that could harm the younger generation. Then the second is Global solidarity, which is between nations, where less fortunate countries must get the attention of other nations. As a clear example, there should not be a stark disparity among the world's nations in terms of vaccine distribution. The last is intergroup solidarity, namely the emergence of various forms of new stigma in society, which is a challenge in building solidarity between groups. This stigmatization affects mental health and wellbeing and makes disease control more complicated.

### 1.2. Aim and research questions of the present study

The background of this research was to find out how social solidarity exists in rural and urban communities during a pandemic. By understanding the phenomenon of social solidarity in society, the policies taken to address the problem will be more effective. Several researchers (Brown, 2020; Angaw, 2021; Stok et al., 2021; Taliep et al., 2021; Kaup et al., 2022) who researched solidarity during a pandemic, it can be concluded that solidarity during a pandemic is dynamic and perspectives also vary. Both in terms of the meaning of solidarity itself and its implementation based on time and place. Therefore, we hypothesize that the manifestation of social solidarity between people living in rural and urban areas will differ in a pandemic situation. The objectives of the research are: (1) to find out the existence of social solidarity in rural and urban communities during the pandemic; (2) to know the different levels of social solidarity in rural and urban communities; and (3) to know the level of community exposure to COVID-19 in rural and urban areas.

The structure of this manuscript is divided into several sections. The first part describes the introduction and research background. Then in the next section an explanation of the method used. The third part describes the characteristics of the respondents, the correlation between variables, and the research units. The next section is a discussion regarding research findings and implications. The last part is the conclusion. In this research, social solidarity is seen before and during the pandemic.

There are several instruments that become research units to answer the three research objectives. First, *gotong royong* to see the characteristics of mutual aid. Second, marriage *banjar* which assesses aspects of solidarity in weddings. The third part then enters during the pandemic. This section discusses attitude in working together during the pandemic. Fourth, willingness to help each other. Fifth, sharing of Information about the prevention and handling of COVID-19. The lastly, number exposed to COVID-19 on Lombok Island.

#### 1.2.1. Materials and methods

**Figure 1:**
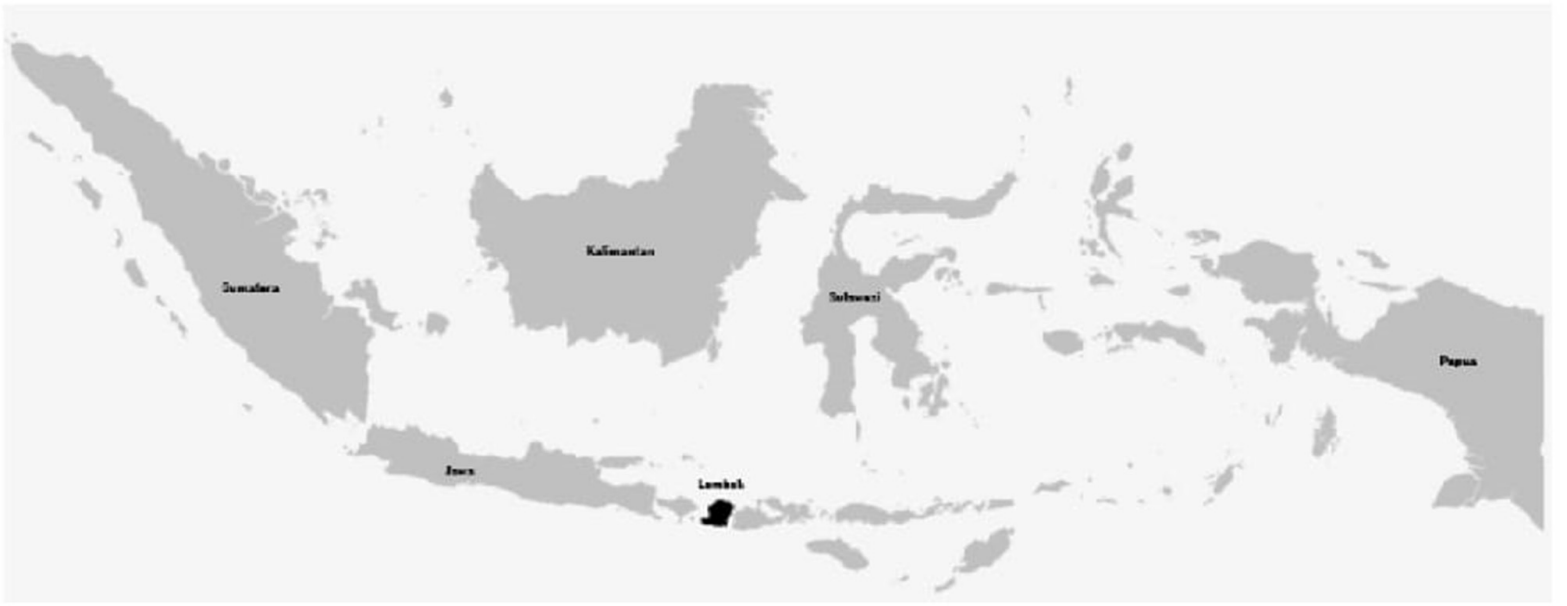
Map of Indonesia. Source: Indonesia and Lombok Island with black dots as research areas; https://www.nicepng.com/ourpic/u2w7ilq8e6ila9w7_peta-indonesia-high-resolution-indinesia-map-vector/ (last accessed, February 20, 2023).

Lombok Island (Figure 1) was chosen as the research location because this island has unique characteristics. *First*, the level of population density is high, but includes a mix of urban and rural communities. *Second*, Lombok is inhabited by people with differing social and economic backgrounds. This research adopted a quantitative approach to identify and analyse the existence of social solidarity in rural and urban communities during the COVID-19 pandemic. A set of questionnaires was distributed and shared by enumerators with 1,100 targeted-respondents from 5 (five) districts/municipalities around Lombok Island in the province of West Nusa Tenggara. The survey was carried out from 14th October to 28th October 2021, that is, during a recovery period from the pandemic while restrictions implemented by the government were still in full force.

In determining the sample size representing the population, the calculation procedure used a random proportional sampling technique (Sugiyono, 2012). In calculating the sample size for each district, adjustments were made based on the characteristics of respondents aged 17 years and over so that the number of respondents for each district would be reflective of the age distribution of the district itself. First, the research population was divided by regencies and municipalities on the island of Lombok, which has 220 villages and towns. The total population of the island is 3,758,631, with 429,651 in Mataram Municipalities, 247,400 in the North Lombok Regency, 721,481 in West Lombok, 1,034,859 in Central Lombok, and 1,325,240 in East Lombok. The total population was determined by the total number of respondents, which was 1,100. This resulted in 126 from the Mataram Municipality, 72 from North Lombok, 200 from West Lombok, 312 from Central Lombok, and 391 from East Lombok. The number of respondent from rural areas was 918 (83.45%) and urban area was 182 (16.54%). These sample sizes are reflective of the number of people living in rural and urban areas on Lombok Island, 88.57, and 11.43%, respectively (NTB Central Bureau of Statistics, 2020).

One way to define solidarity is as any action that increases people's welfare at the local or community level (Paskov and Dewilde, 2012). However, the concept of social solidarity does not stand alone. It relates to other social concepts, such as social cohesion, social trust, social capital, and the distribution of various resources to meet the needs of people. Social solidarity is also related to social construction of social relations, values, and group identity (Berman and Philips, 2004). Hence, there are 5 (five) indicators of social solidarity measured in this study, namely:

1. An attitude of cooperation among citizens, which in Indonesia is known as *gotong royong;*
2. Participation in preparations for the marriage *banjar*;
3. An attitude of working together or cooperating with others during the pandemic;
4. A willingness to help others in the form of donations during a pandemic, that is, of mutual support; and
5. A desire to remind each other to comply with various health protocols during the pandemic, that is, a willingness to share information on a variety of issues, including information that would be useful for preventing the spread of COVID-19.

*Gotong royong* and *banjar* are two traditional institutions that we use as indicators of social solidarity unique to the people of Lombok. *Gotong royong* is a kind of mutual assistance that reflects genuine indigenous notions of moral obligations and generalized reciprocity; it is contextualized to build social solidarity in handling COVID-19 and manifested by the active participation of each individual to provide added or a positive value to each object, opposition, or needs of many people around them (Sultan and Rapi, 2020; Muqsith et al., 2021; Artayasa et al., 2022; Shin et al., 2022). At the same time, *banjar* can be defined as a form of small and limited community association or group in which many social activities take place or local wisdom as well as part of the social system of society which has been maintained regarding beneficial impacts for networked individuals (Jamiluddin, 2022; Wijayanti et al., 2022).

For each of those indicators, alternative answers that indicate the level of desire to participate are prepared. This study employed a Likert scale of 1–5, where one is very low, two is low, three is moderate, four is high, and five is very high. Likert scale is a type of scale frequently used to measure perceptions, attitudes, and opinions for the purposes of statistical analysis. Thus, it is hoped that an overview of the level of social solidarity in the community will be obtained, which is the unit of analysis of this research. According to Sugiyono (2018), a Likert scale is appropriate to measure views or perceptions of a person or group of people so that the researcher can obtain an accurate picture of the social phenomena being studied.

Chi square was used to test whether there were differences in social solidarity between those who lived in rural areas and those who lived in urban areas. The chi-square test is often used in research that examines the relationship of two variables (Sharpe, 2015). Chi-square is an analytical technique to determine the difference in the frequency of observations from the frequency of expectations based on a random distribution of paired cases.

Meanwhile, to determine the number of people who were exposed to and died from COVID-19 in rural and urban areas on Lombok Island, we used data released by the West Nusa Tenggara Province COVID-19 Task Force. The data we collect is data that had occurred since the outbreak of COVID-19 in early 2020 until the end of 2021 when this research was conducted.

## 2. Results

### 2.1. Respondent characteristics

The distribution of respondents by gender can be seen by comparing the number of male and female respondents; the difference is very thin (Table 1). For example, it was recorded that the male respondents were 50.9% of the 1,100 respondents, while the female respondents were 49.1%. From the characteristics of the respondent's area of residence, out of 1,100 respondents, it was recorded that 83.5% of the respondents resided in rural areas and 16.5% of respondents lived in urban areas. Then, there is a grouping of respondents based on age. The distribution of number of respondents based on their age level was grouped into several groups. Based on data from 1,100 existing respondents, from the most to the least, of the 25.8% of respondents aged 35–44 years, 24.4% of respondents aged 25–34 years, 20.4% of respondents aged 45–54 years, 14.8% of respondents aged 17–24 years, and 10.7% of respondents aged 55–64 years. Meanwhile, only 3.8% of respondents were 65 years and over, and 0.1% of respondents from the age group of fewer than 17 years were married.

**Table 1:** Respondent characteristic.

**Respondent characteristics**	**Percentage**
**Gender**	
Male	50.9%
Female	49.1%
**Residence**	
Rural	83.5%
Urban	16.5%
**Age**	
<17 years old but married	0.1%
17–24 years	14.8%
25–34 years	24.4%
35–44 years	25.8%
45–54 years	20.4%
55–64 years	10.7%
65 years and over	3.8%
**Level of education**	
Never went to school	4%
Did not finish elementary school	8.1%
Graduated from elementary school	14.5%
Graduated from high school	19.6%
High school graduate	40.8%
Diploma (D1-D2-D3)	1.8%
Bachelor (S1/D4)	11%
Postgraduate (S2-S3)	0.2%
**Occupation**	
Farmers/breeders (owners/tenants)	16.4%
Fisherman (owner)	1.1%
Laborers (teners/fishermen/construction/masters)	18.5%
Small traders (bakulan, street vendors, stalls, etc.)	29.2%
Private/Professional employees (private doctors, lawyers, etc.)	4.7%
Entrepreneurs/businessmen/big traders	12.9%
Civil Servant/Teacher/Lecturer/TNI/Polri	5.1%
Non-Civil Servant employees (honorary teachers/honorary employees, non-permanent teachers, etc.)	6.7%
Other	5.4%
Doesn't work (housewives, students, retired person, etc.)	34.0%

Sources: data of the study calculated (2022).

The diversity of educational levels of the 1,100 respondents was mainly in the category of graduating from high school/equivalent, namely 40.8% of respondents. However, few respondents were included in the category of never going to school, as much as 4%. Nevertheless, the data shows that the education level of respondents is still relatively low because as many as 8.1% of respondents did not finish elementary school/equivalent, 14.5% of respondents graduated from elementary school/equivalent, and 19.6% of respondents graduated from junior high school/equivalent. On the other hand, the rest shows that some respondents can continue their education to a higher level, namely Diploma (D1-D2-D3) with 1.8% of respondents, Bachelor (S1/D4) with 11% of respondents, and Postgraduate (S2-S3) with 0.2 % of respondents.

Based on their main daily activities, out of 1,100 respondents, 34% of respondents said they do not work, including those who have been in school, are housewives, and are retired person. Meanwhile, respondents who work as the main activity are divided into several types of work. As many as 29.2% of respondents worked as small traders, 18.5% of respondents as laborers, 16.4% of respondents as farmers/breeders, 12.9% of respondents as entrepreneurs, and the rest relied on a living from work such as non-ASN employees, ASN, private employees, fishermen, and others.

### 2.2. Correlation between variables

According to Taylor (2019) and Agung (2020), there are three interrelated elements that help with an understanding about how a pandemic like COVID-19 affects a society; namely, the virus itself and characteristics associated with its transmission and its physical effects on people; the psychological element of people who feel threatened by this pandemic; and the environment in which they live, including both its physical and sociological dimensions. In terms of the environment in which they live, villages and cities are important areas to study what phenomena occur in each in terms of the first two elements. Is the influence of location important to the spread of the COVID-19 pandemic because its influence extends not only to individuals or families but also to communities and groups at various levels? Are there differing levels of concern about the effects of COVID-19 between those living in urban and rural communities, and if so, how do these concerns shape the patterns of their daily living?

The following describes the results (Table 2) of the research we have conducted on people, divided into two locations, namely rural and urban areas. Within each type of community, we look at possible differences in social solidarity, which we measure with five indicators, namely: (1) *gotong royong*, (2) marriages *banjar*, (3) an attitude of cooperation, (4) mutual assistance, and (5) sharing of information.

**Table 2:** Research Indicators for social solidarity in rural and urban areas.

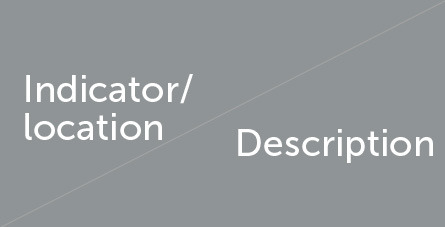	**Don't know/no answer**	**Very low**	**Low**	**Moderate**	**High**	**Very high**	**Total**	***P*-value**
**1**. ***Gotong-royong***								
Rural	1.7%	2.5%	11.1%	27.1%	31.5%	26.0%	100%	0.000
Urban	1.6%	14.8%	14.3%	28%	28.6%	12.6%	100%	
**2. Marriage** ***banjar***								
Rural	2.5%	1.0%	5.9%	23.0%	33.9%	33.8%	100%	0.000
Urban	1.1%	13.2%	8.8%	31.3%	29.1%	16.5%	100%	
**3. Cooperation**								
Rural	1.7%	1.4%	8.6%	28.0%	36.5%	23.7%	100%	0.000
Urban	0.0%	13.2%	16.5%	31.3%	26.9%	12.1%	100%	
**4. Mutual assistance**								
Rural	4.8%	6.6%	10.7%	31.9%	29.0%	17.0%	100%	0.000
Urban	1.6%	13.2%	24.7%	25.8%	18.1%	16.5%	100%	
**5. Sharing of information**								
Rural	3.7%	3.2%	11.9%	24.4%	33.4%	23.4%	100%	0.000
Urban	1.6%	17.6%	13.2%	31.9%	21.4%	14.3%	100%	

Resource: Study analysis by authors, (2021).

#### 2.2.1. Gotong royong

From a Durkheimian point of view, contextually, rural and urban areas have distinctive characteristics. The findings in this study also show that during the pandemic, when this research was conducted, social solidarity in the two regions with the variable gotong royong displayed differences. The Table 2 shows these dynamics in terms of the enthusiasm for cooperation among respondents from both rural and urban communities during the pandemic. From the chi-square, it can be concluded from the *p*-value that there was a significant difference in *gotong royong* or cooperative activities during the pandemic between rural and urban communities. Enthusiasm of people for implementing *gotong royong* in rural areas was higher than in urban areas. This implies that people in rural areas were less observant of prohibitions on gathering and maintaining distance during the pandemic. That is why cooperative activities such as places of worship and public facilities such as roads and public market, during the pandemic period, did not experience slowdowns, especially in rural areas. Violation of the prohibition on social distancing shows the limitation of weak sanctions and the monitoring of health protocol policies in rural areas. In a similar study, Mishra and Rath (2020) advocate a contextual approach to health prevention by emphasizing the roots of social solidarity at the local level to devise acceptable methods to prevent the spread of future pandemics. In Indonesia, *gotong royong* is the central to the culture's collective consciousness that defines solidarity and how people cooperatively act, both physically and spiritually (Hanif, 2021).

#### 2.2.2. Marriage *banjar*

The wedding reception is an event that has become a tradition in Indonesia, both those who live in rural areas and urban areas. However, these events are strictly limited by the number of guests, and even banned altogether, with the onset of the COVID-19 pandemic. Therefore, making the marriage *banjar* an indicator of social solidarity is essential to understanding rural-urban differences in the context of this pandemic. Respondents from rural communities were more likely to believe that participation in marriage *banjar* was important, even during times of COVID-19 because it is an indicator of social solidarity. *Banjar* is a traditional institution for a special purpose.

Meanwhile, respondents from urban communities for the marriage *banjar* variable only reached 45.6% (“high” or “very high”). In rural communities, respondents who answered “high” or “very high” stood at 67.7%. This shows that rural communities have a higher level of attachment to mingling and being involved in the marriage reception or *banjar* during the pandemic. The chi-square analysis also obtained a *p*-value that indicates that the difference in social solidarity between rural and urban areas was significant. In other words, it can be said that although there is a government prohibition related to the implementation of marriage *banjar* activities, people in rural areas were more likely to ignore them. They were more concerned with the social solidarity shown by their participation in *banjar* than their adherence to the health protocols set by the government.

#### 2.2.3. Attitude in working together during the pandemic

This indicator of solidarity was measured based on attitudes that encourage individual actions to help others, such as borrowing money or goods and visiting sick neighbors. In general, it can be concluded, based on the results in Table 2, that there were significant rural-urban differences. In rural areas, people generally had a greater concern, even during the COVID-19 pandemic about the importance of working together. In rural communities, respondents who answered “high” or “very high” stood at 60.2%. In contrast, the percentage from the same two categories for respondents from urban communities was only 39.0%. Again, the difference in the percentage of high and very high standards between people in rural areas and urban communities shows the difference in their adherence to health protocols, especially in terms of maintaining recommended distances. The chi-square value indicates a *p*-value of 0.00, which means the difference was statistically significant. For people in rural areas, there was a greater need to interact with each other to meet daily needs, and in terms of visiting those who are sick, they tended to ignore health protocols. In other words, government policies related to health protocols for rural areas cannot be implemented as effectively as in urban because of differences in social solidarity.

#### 2.2.4. Willingness to help each other

In this indicator of solidarity, the willingness to help each other is measured based on donations of money or goods. Like the previous two indicators, there was again a significant difference between the attitudes of respondents from rural and urban communities in the effort to set aside money and goods for social donations. Based on the data in Table 2 it can be seen that those who indicated either “high” or “very high” in rural communities was 46.0%, while people in urban areas added up to 34.6%. Although this difference is not as large as the previous two indicators, the chi-square analysis was still statistically significant with a *p*-value of 0.00. Again, it means that the social solidarity of respondents living in rural areas is higher than those from urban areas.

Health issues during the pandemic are not only based on physical health, but also has many social issues. Research Egcas et al. (2021) shows that mental health is a topic that was quite intensely discussed during the pandemic. This issue is connected with the level of community welfare during the pandemic. Efforts to help each other form a type of defensive social ecology in terms of financial and mental health. The impact of the pandemic on the economy also supports findings related to the actuality of mutual assistance carried out by the community during the pandemic. In their research, Nguyen et al. (2021) showed a significant pandemic effect on the global economy, which includes manufacturing, education, finance, pharmaceuticals, aviation, tourism, and food.

#### 2.2.5. Sharing of information

The indicator of willingness to share information included shared knowledge on market prices for agricultural products, developments in agricultural technology, and detailed information on government policies related to the pandemic. For information-sharing indicators, respondents from rural areas who answered “high” and “very high” was 56.8%. Meanwhile, for the same indicator, it only reached 35.7% for respondents from urban communities. This is understandable considering that in rural communities dominated by agricultural activities, the need for sharing information should be much higher than in urban communities. The tradition of informing each other about the process and means of production and marketing of agricultural products has been long-established and is not easily influenced by external factors. From the results of this study, for example, the existence of a pandemic did not dampen the enthusiasm and motivation of farmers and other rural residents to keep sharing information. The solidarity of rural residents that has been built over the many generation is also useful for sharing current information related to the pandemic and to associated government policies. According to Jamal et al. (2009), other factors that influence sharing include the availability of information in the form of brochures and other educational materials about health protocols and the extent of publicity about the threat and impact of COVID-19 in the community.

#### 2.2.6. Number exposed to COVID-19 on Lombok Island

The next part of this article is related to the number of those exposed to COVID-19. From the data released by the West Nusa Tenggara Province COVID-19 Task Force, until the end of 2021, when this research was conducted, the number of those exposed can be seen in Table 3. This is in line with the data released by the National COVID-19 Task Force (Nugraheny, 2020), which are categorized as rural areas (rural) are those who come from the regency area. In comparison, those from the municipality are categorized as cities (urban). Comparison of data in rural areas and urban areas can be seen in Table 3.

**Table 3:** The number of those exposed to COVID-19 in rural and urban areas throughout Lombok Island until December 2021.

**Area**	**Exposure**	**Percentage (%)**	**Died**	**Percentage (%)**	**Death rate**
Urban	6705	41	254	43	3.79
Rural	9640	59	337	57	3.49

Resource: West Nusa Tenggara Province COVID-19 Task Force (2022).

Lombok Island is an area in Indonesia with a relatively high population exposed to COVID-19, including the number who died. From the data in Table 2, it can be concluded that the number and percentage exposed in rural areas are relatively higher compared to those in urban areas. From the percentage level, it can be concluded that the difference between rural and urban areas is quite significant (41 and 59%). However, from the fatality rate, the percentage in rural areas is lower than in urban areas (3.49 and 3.79%).

## 3. Discussion

Durkheim viewed changes wrought by the industrial revolution in Europe as a reference for understanding differences between more traditional societies and those that were industrializing (Albrow, 2013; Hanifah, 2019). Durkheim (2019) explained that social solidarity is a state of the relationship between individuals or groups based on shared morals and beliefs and that is strengthened by shared emotional experiences. In line with changes in interaction patterns that occur due to the pandemic, it will also encourage the escalation of cooperation. People living in communities of different sizes will work more hand-in-hand in planning and overcoming phenomena like pandemics.

The five indicators measured in this study (*gotong royong*, enthusiasm for marriage *banjars*, cooperation, willingness to help each other, and sharing of information among residents), show that social solidarity in rural and urban areas on Lombok Island is relatively different. The five indicators can be divided into three types in order to clarify the discussion. The first type is related to gotong royong and marriage *banjar*, which can be described as community-based social activities and events (local social gatherings). Based on the analysis and description above (Table 2), we see that social solidarity in terms of indicators of social solidarity is significantly different between those living in villages compared to those in the city. This reality implies that various social and cultural activities that provide opportunities for people to gather in rural areas were still carried out, despite advice to the contrary by governmental entities (Derung, 2019). On the other hand, in urban areas, cooperation as an indicator of social solidarity was weaker. According to Muqsith et al. (2021), this situation is because urban people's awareness and understanding of the dangers of COVID-19 was higher than for rural people. Weaker law enforcement against health protocol violations in rural areas was another contributing factor that enables their ability to sustain cooperative activities during the pandemic.

The second type is mutually beneficial cooperation, a combination of a willingness to cooperate and a desire to help others during a pandemic. From the analysis described previously, it can be concluded that there were significant differences between rural areas and urban areas. This conclusion implies that the behavior of people in village communities in terms of working together and helping others during the pandemic has not changed much, or not at all. In other words, the existence of a pandemic, along with various government policies, does not dampen people's enthusiasm in rural areas to work together and to help each other, maintaining the social solidarity of rural communities despite the challenges of COVID-19 and preventive measures. A campaign by fellow citizens to comply with the health protocols is a form of citizen effort to jointly fight this COVID-19 (Gunasekaran et al., 2020; Szczesniak et al., 2020). According to research by Meinzen-Dick (2020) and Valeriani et al. (2020), this social solidarity is indispensable in dealing with a pandemic. With high solidarity, many community members have become very helpful in overcoming the various problems they face, both in terms of their health and the economy. Even in Canada, as reported by the results of a study by Smythe et al. (2021), with high social solidarity, problems in the education sector that were severely affected by this pandemic can then be resolved.

The dilemma that has become the subject of discussion in this research is that the government hopes that the public will comply with the health protocol rules to prevent the spread of the COVID-19 virus quickly. Meanwhile, health protocols, such as maintaining distance and limiting direct contact cause social solidarity in society to decline, as evidenced by this study, occurs more in urban areas than in rural areas. In their article on social solidarity in the pandemic era, Haryadi and Malitasari (2020) stated that this sense of solidarity arises because of empathy for those infected by the COVID-19 virus. The community also appreciates community groups who take the initiative to assist others, especially those who are less fortunate. According to researchers (Mishra and Rath, 2020; Sayuti, 2020), the goal is to increase the community's resilience in facing this pandemic. With the high level of community resilience, the level of community exposure to COVID-19 will also be lower. The higher level of solidarity in rural communities compared to urban areas can be thought to be a contributing factor to the lower death rate of those in rural areas compared to those in urban areas (Table 3). With higher solidarity, efforts to prevent deaths from exposure to COVID-19 can be reduced.

The third type of indicator of social solidarity is the sharing of information. This information-sharing activity is a strong indicator of solidarity because it involves at least two aspects. The first aspect is how people are affected by the pandemic when they have to disseminate information to others on a day-to-day living. For example, farmers sharing of information about production facilities and market prices is very important and affects their economic wellbeing. This traditional pattern of information exchange was continued and even improved in order to disseminate information related to the pandemic (Sayuti and Hidayati, 2021). As described previously, the information-sharing systems that are part of the social fabric of agricultural communities can be utilized to monitor the spread of COVID-19 and how to avoid catching COVID-19, and to treat COVID-19 symptoms if it is acquired. Second, how people can take advantage of existing technology to share of information during a pandemic without reducing the sense of social solidarity among each other becomes a challenge for health care and prevention initiatives by health care organizations and various governmental agencies. According to Muqsith et al. (2021), this information technology is an alternative means that is quite effective for communication between residents because of the prohibitions on leaving the house. Based on the results of this study, it can be concluded that during the pandemic period, there were significant differences between rural and urban areas. Specifically, the results show that the pandemic has affected information sharing activities more so in urban areas than in rural localities. Activities usually carried out directly to and from other community members are limited because urban residents generally adhered to the health protocols more so than people living in rural communities. This means that they keep their distance and avoid crowds as stipulated by the government. According to Zahri et al. (2018), people living in urban communities use various social media to stay in touch and share of information.

Meanwhile, information-sharing type activities still rely on direct relationships and interactions in rural communities more so than in urban communities. Rural communities tend to be more homogeneous than urban communities and rely on primary-type relationships (i.e., mostly face-to-face) as the basis for the glue of their mechanical social solidarity. The economic needs of rural communities on islands like Lombok are highly dependent on the agricultural sector, while urban neighborhoods with more diverse economies tend to be heterogeneous, with communication based on the expertise of the person with whom information is exchanged. Hence, in urban areas that display higher levels of organic solidarity, the pandemic more likely disrupts information-sharing because of the health protocols that the government can more readily enforce in a strict manner.

The need for rural communities to share their resources reflects the pandemic's impact, which then results in greater income inequality, a widening health crises, and ultimately causing limited access to economic opportunities beyond agriculture. Physical distance restrictions on all indicators of social solidarity are translations for the ways people conduct their lives on a day-to-day basis (Paskov and Dewilde, 2012). The findings of this study provide important information regarding the characteristics of rural social solidarity, which are relatively different from the character of social solidarity in urban areas during the pandemic. Whether these findings will also occur when the pandemic has passed, will require further study, shedding light not only on how to respond to pandemics, but on the fundamental sociological character of rural and urban communities in contemporary times.

### 3.1. Implications for future policies

The occurrence of a worldwide pandemic affecting every community on earth has shown how geographical distances are becoming less relevant and the shadow of globalization shows us the reflection of its impact on the daily lives of people everywhere (Mas-Coma et al., 2020; Osotimehin and Popov, 2020). The pandemic also shows both communal and individual expressions of social solidarity among members of both rural and urban communities. In line with Reichlin's (2011) view, social solidarity has the potential to unite universal morality to the needs of humanity. Both Durkheim's and Weber's reflection on social solidarity refers to the intimacy of a community group with its members and vice versa (Johnson, 1994; Ritzer, 2012). The emphasis of this argument lies on social cohesion, which is fostered using collective values. The findings from this study open up more critical questions for future policy development in at least two main areas, namely health and the economy. The health crisis has disrupted economic activities, becoming the basis for evidence of how inequality is expressed in rural and urban communities.

From a policy-making point of view, Durkheim emphasized the importance of law as a guiding compass for constructing social solidarity. Classification of law in Durkheim's view is divided into repressive and restitutive. The repressive rule refers to collective sanctions, while restitutive is attached to sanctions for violations. From a health perspective, preventing the spread of the epidemic using both methods is still relevant for COVID-19 (Fisher and Wilder-Smith, 2020; Tiffany, 2020). Various policies that insulate physical and social distance to mitigate the spread of COVID-19, such as the analysis in this study, shows that normative and affective compliance have not been clearly defined. Physical and social distancing based on government regulations is likely to be more inconsistent with the social solidarity of rural people than urban people, that is, requires a bigger adjustment to their lifestyles. Hence, social solidarity in this study plays a vital role as a scientific basis for further empirically based studies on social resilience and the fine-tuning of policy agendas. From this viewpoint, it can be seen that policies implemented in urban areas cannot necessarily be applied to people in rural areas in the same manner. Indeed, local context will influence the effectiveness of policies related to health and many other areas as well.

The future challenge is formulating inclusive but effective policies amidst the diversity of forms of social solidarity found in rural and urban settings. Furthermore, how is the communication strategy regarding the substance of the approach taken so that it can be implemented by all community members, both those who live in rural and those who reside in urban areas? From this research, it can be seen that not all existing policies can be implemented. The policy of social distancing, for example, is more difficult to implement in rural areas. Hence, this and other restrictions where the policy does not pay attention to the location of implementation will less likely be successful. Policies between rural and urban areas are generally not differentiated. Therefore, a location-specific policy formulation is needed so that if there is a failure in its implementation, the improvement of the formulation is also location-based (Sayuti et al., 2021). This means that we should not assume the conditions of one community are the same as another, whose socio-cultural patterns may be different. People in rural areas with greater mechanical solidarity and urban communities with greater organic social solidarity should receive locality-adjusted treatments because their needs and demands are also distinctive.

What is needed is collective awareness from various levels of society without exception, including policymakers in the government. The rural-urban differences in social solidarity require more in-depth research. One way to go about this kind of research is to ask how social solidarity at different kinds of places is influenced by and in turn influence things like a COVID-19 pandemic? This research provides only a glimpse at the ways the context of local places can affect the implementation of various policies during a pandemic so that they can be improved. The number of exposed and the rate of mortality could be minimized if the policies were more locality-oriented, and not simply one-size-fits-all guidelines applied uniformly and often ineffectively to diverse places.

## 4. Conclusion

This research found that the existence of social solidarity in both rural and urban communities during the pandemic. The level of social solidarity in rural areas is higher than in urban areas. While related to the number of those who were exposed and died, from the data released by the Provincial COVID-19 Task Force, conditions were higher in rural areas compared to the number of those who were exposed or who died in urban areas. However, in terms of the death rate, the level of death rate in urban areas is higher than in rural areas. This condition indicates differences in the characteristics of rural and urban communities, which can be considered in implementing policies during a future pandemic. This research provides evidence for local governments in formulating policies with a social solidarity perspective by taking into account the different characteristics of rural and urban communities.

The existence of the COVID-19 pandemic that has occurred in almost all countries has raised awareness that their level of resilience, in terms of such sociological dynamics as social solidarity, is variable. When dealing with this pandemic, attitudes or behaviors also vary according to their educational, socio-cultural background, and especially the area where they live. Therefore, by the findings of this study, we can suggest several things. First, public awareness must be improved that social solidarity must still be maintained in the face of the COVID-19 pandemic. The second suggestion is that policies related to the pandemic or other procedures in dealing with extraordinary phenomena like this must pay attention to the socio-cultural character and the location of the community's residents. The third suggestion is related to further research. Research on social solidarity needs to be repeated in the post-pandemic period. It is necessary to know whether the current study results are different or will remain the same when the research is carried out after the pandemic no longer exists.

### 4.1. Permission to reuse and copyright

Third-party figure material in this work is used in a fair way, and the copyright holders have noted, it “Can be used for the creative projects.” in their website Therefore, third-party figure material can be used without requiring certain permissions.

## Data availability statement

The raw data supporting the conclusions of this article will be made available by the authors, without undue reservation.

## Ethics statement

Ethical review and approval was not required for the study on human participants in accordance with the local legislation and institutional requirements. Written informed consent from the participants' legal guardian/next of kin was not required to participate in this study in accordance with the national legislation and the institutional requirements.

## Author contributions

RS, MT, and MM contributed to conception and design of the study. SH organized the database and performed the statistical analysis. RS wrote the first draft of the manuscript. AE wrote sections of the manuscript. All authors contributed to manuscript revision, read, and approved the submitted version.
